# Is Fluorescence Valid to Monitor Removal of Protein Bound Uremic Solutes in Dialysis?

**DOI:** 10.1371/journal.pone.0156541

**Published:** 2016-05-26

**Authors:** Jürgen Arund, Merike Luman, Fredrik Uhlin, Risto Tanner, Ivo Fridolin

**Affiliations:** 1 Department of Biomedical Engineering, Technomedicum, Tallinn University of Technology, Tallinn, Estonia; 2 Department of Dialysis and Nephrology, North Estonia Medical Centre, Tallinn, Estonia; 3 Department of Nephrology, Region Östergötland, and Department of Medical and Health Sciences, Faculty of Health Sciences, Linköping University, Linköping, Sweden; CNR, ITALY

## Abstract

The aim of this study was to evaluate the contribution and removal dynamics of the main fluorophores during dialysis by analyzing the spent dialysate samples to prove the hypothesis whether the fluorescence of spent dialysate can be utilized for monitoring removal of any of the protein bound uremic solute. A high performance liquid chromatography system was used to separate and quantify fluorophoric solutes in the spent dialysate sampled at the start and the end of 99 dialysis sessions, including 57 hemodialysis and 42 hemodiafiltration treatments. Fluorescence was acquired at excitation 280 nm and emission 360 nm. The main fluorophores found in samples were identified as indole derivatives: tryptophan, indoxyl glucuronide, indoxyl sulfate, 5-hydroxy-indoleacetic acid, indoleacetyl glutamine, and indoleacetic acid. The highest contribution (35 ± 11%) was found to arise from indoxyl sulfate. Strong correlation between contribution values at the start and end of dialysis (R^2^ = 0.90) indicated to the stable contribution during the course of the dialysis. The reduction ratio of indoxyl sulfate was very close to the decrease of the total fluorescence signal of the spent dialysate (49 ± 14% vs 51 ± 13% respectively, P = 0.30, N = 99) and there was strong correlation between these reduction ratio values (R^2^ = 0.86). On-line fluorescence measurements were carried out to illustrate the technological possibility for real-time dialysis fluorescence monitoring reflecting the removal of the main fluorophores from blood into spent dialysate.

In summary, since a predominant part of the fluorescence signal at excitation 280 nm and emission 360 nm in the spent dialysate originates from protein bound derivatives of indoles, metabolites of tryptophan and indole, the fluorescence signal at this wavelength region has high potential to be utilized for monitoring the removal of slowly dialyzed uremic toxin indoxyl sulfate.

## Introduction

A wide range of uremic solutes retain in the bodies of patients with end-stage chronic kidney disease. According to the EuTOX workgroup classification, these uremic solutes can be divided into three groups: free water-soluble low-molecular-weight solutes (small molecules), protein-bound solutes (PBS) and middle molecules (MM) [1]. During dialysis, removal kinetics of these groups of uremic solutes is different. In general, the removal of PBS, for example indoxyl sulfate (IS), and MM is slower than that of representatives from the group of small uremic toxic molecules like uric acid, and classical markers of urea and creatinine [2–4]. Introduction of convective dialysis variants such as hemodiafiltration has improved the elimination of MM but the difficulty consists of PBS [5]. Although the classical markers, urea and creatinine are representative for the removal of the group of small molecules, they give no indication to the other cofactors of mortality of patients with chronic kidney disease [6], such as the role of PBS to the mortality rate [7]. Besides the urea based Kt/V, a dialysis dose parameter for the small water soluble molecules, there are no established markers to assess the dialysis dose for the removal of PBS. Among the PBS, the highest levels of binding to the proteins has been shown for IS, p-cresyl sulfate and 3-carboxy-4-methyl-5-propyl-2-furanpropionic acid (CMPF) [8], all considered as uremic toxins. The most widely studied solute among those protein-bound uremic toxins is IS, a metabolic end product of indole originating from gut flora [9, 10]. The biologic and/or toxic effects of IS leading to cardiovascular mortality and renal impairment have been reported in several studies [11].

The *de facto* most widely used method for PBS determination is optically with a diode array detector or a fluorescence detector by HPLC, since fluorescence offers a highly selective and sensitive analytical method for detection and quantification [12]. However, the HPLC analysis is time-consuming and requires a laboratory with sophisticated analytical chromatography systems and skilled personnel. Thus, it is difficult to monitor levels and removal of PBS in large scale studies and during routine daily dialysis. Novel and robust on-line monitoring methods, similar to those established for urea based dialysis dose optical estimation [13], would be beneficial.

Although the usage of fluorescence has been suggested as a medical diagnostic tool from the beginning of the discovery of the fluorescence phenomenon in uremic fluids [14, 15], fluorescence has not been implemented for the analysis of spent dialysate without the use of HPLC. Lately, a novel optical method was proposed to monitor the concentration and the removal of protein bound uremic solute of IS in the spent dialysate by utilizing fluorescence without the need for HPLC [16]. However, origin of the fluorescence of spent dialysate by means of identification and contribution of single fluorophores to total fluorescence of spent dialysate remain still elusive.

The aim of this study was to investigate the contribution and removal dynamics of the main fluorophores during dialysis by analyzing the spent dialysate samples to prove the hypothesis whether the fluorescence of spent dialysate can be utilized for monitoring removal of any of the protein bound uremic solute.

## Materials and Methods

### Clinical study

The study was performed after approval of the protocol by the Regional Ethical Review Board, Linköping, Sweden decision on application no. M153-07 (10.10.2007) and by the Tallinn Medical Research Ethics Committee at the National Institute for Health Development, Estonia decision no. 2349 (issued 15.03.2011). A written informed consent was obtained from all participating patients. The study included 20 patients from Tallinn (10 male, 10 female, 59±12 years) and 8 patients from Linköping (7 male, 1 female, 73±14 years). Patients were followed in total 99 sessions from which 57 were hemodialysis (HD) and 42 hemodiafiltration (HDF) sessions. The dialysis machines used were Fresenius 5008 (Fresenius Medical Care, Germany). The dialyzers were FX8, FX10, FX80, FX100, FX800, and FX1000 (Fresenius Medical Care, Germany). The duration of the treatments varied between 180 to 270 minutes, the dialysate flow was 500 ml/min and the blood flow varied between 250–350 ml/min.

**Fig 1:**
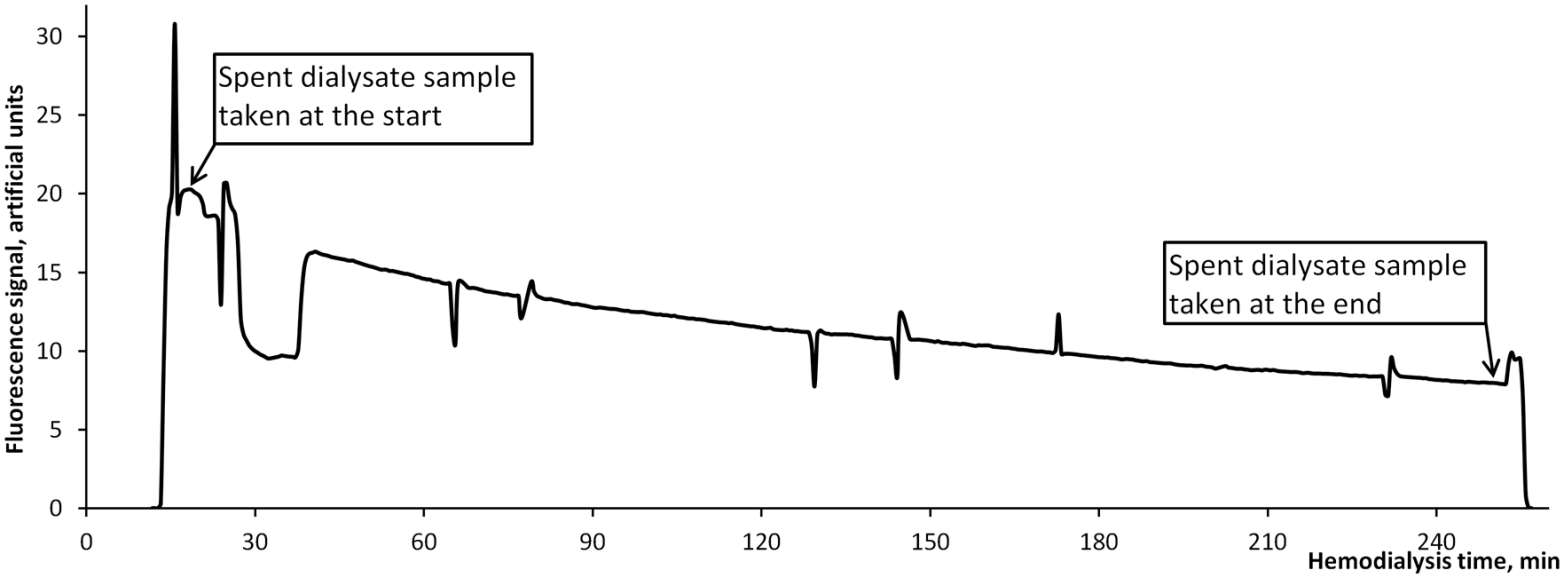
Example of a fluorescence signal of spent dialysate monitored online during a hemodialysis session.

During the dialysis, the following dialysate samples were taken: 1) 10 minutes after the start of the dialysis session; and 2) at the end of the treatment (Fig 1). Sampling at the moments of self-tests of the dialysis machine was avoided. Pure dialysate was collected as the reference solution before the start of a dialysis session, when the dialysis machine was prepared for startup and the conductivity was stable.

In parallel to dialysate sampling, on-line fluorescence measurements were carried out during nine selected dialysis sessions (Fig 1) to illustrate technological possibility for real-time dialysis fluorescence monitoring. The clinical set-up was similar to the on-line UV-absorbance measurements as reported earlier [17]. On-line fluorescence measurements were performed by a spectrofluorometer FLD3400RS (Dionex, Sunnyvale, USA) with a fluorimetrical flow cuvette connected to the drain outlet of the dialysis machine. Partial flow of the spent dialysate from the main drain tube was guided into the flow cuvette with a separate pump LC-9A (Shimadzu, Kyoto, Japan), providing a dialysate flow of 2 mL/min. The on-line fluorescence was measured at the excitation (Ex) wavelength of 280 nm and the emission (Em) wavelength of 360 nm with the sampling rate of 12 measurements per minute.

### HPLC Study

All dialysate samples were acidified down to pH 4.25 with formic acid before the HPLC analysis for the sake of stable chromatographic retention times. The HPLC system consisted of a quaternary gradient pump unit, a thermostated autosampler, a column oven, a diode array spectrophotometric detector (DAD), a fluorescence detector (FLD), all Ultimate 3000 Series instruments from Dionex (Sunnyvale, CA, USA), and two continuous columns of Poroshell 120 C18 4.6 x 150 mm from Agilent Instruments (Wilmington, DE, USA) with a security guard KJO-4282 from Phenomenex (Torrance, CA, USA). The samples were kept at 6°C in the autosampler. The eluent was mixed with 0.05 M formic acid adjusted to pH 4.25 with ammonium hydroxide (A), and organic solvent mixture of HPLC grade methanol and HPLC-S grade acetonitrile, both from Rathburn (Walkerburn, Scotland) in the ratio of 9:1 with 0.05 M ammonium formiate salt (B). The three-step linear gradient elution program was used, as specified in Table 1.

**Table 1:** HPLC gradient program.

Step	Time (min)	Buffer (A) %	Organic solvent (B) %	Curve type
0	0	99	1	
1	6	99	1	5
2	39	10	90	7
3	15	10	90	5

The total flow rate of 0.6 mL/min was used at the column temperature of 40°C. All the spent dialysate samples were analyzed by recording 2-dimensional (2D) fluorescence acquisition chromatogram at the Ex: 280 nm and Em: 360 nm with a measurement interval of 0.5 s. For all 2D chromatograms, a blank run was subtracted from the signal. The 3-dimensional (3D) fluorescence acquisition chromatograms were recorded for 10 selected spent dialysate samples taken at the start of the dialysis, which had the highest common characteristic peak intensities in 2D chromatograms. For 3D chromatograms, each sample was chromatographed two times: one for fluorescence excitation scan and the other for fluorescence Em scan. The fluorescence scanning parameters are given in Table 2 at the measurement interval of 0.5 s. The chromatographic data was processed by Chromeleon 7.1 software by Thermo Scientific (Waltham, USA).

**Table 2:** Chromatogram recording program.

		Excitation	Emission	Samples
**2D acquisition**		280 nm	360 nm	All
**3D acquisition**	Excitation scan	220…340 nm	360 nm	10 selected
	Emission scan	280 nm	300…500 nm	10 selected

For mass spectrum analysis, a quadrupole time-of-flight mass spectrometer micrOTOF-Q II with an ESI source was used (Bruker, Billerica, USA). For both negative and positive ion mode, the parameters for sample analysis were as follows: mass range of m/z 50–700; ion source temperature of 200°C, ESI voltage of 4.5 kV, ESI nebulization gas flow of 8.0 L/min, drying gas flow of 1.2 bar, detector voltage of 2.03 kV, acquisition rate of 1 Hz. Mass calibration was carried out using a sodium formate solution (10 mmol/L) from m/z 50 to 700. Data acquisition was performed using software Compass HyStar version 3.2, and processing of the data was carried out with Compass DataAnalysis version 4.0 SP1 (both Bruker, Billerica, USA).

Every peak in the HPLC chromatograms was characterized by the retention time and the mass spectrum. The chromatographic peaks were identified by comparing the retention time and the mass spectrum of the spent dialysate with the reference compound.

In the analysis of the 3D fluorescence chromatograms, the fluorescence Em and Ex spectra of the peaks were calculated by summing all the spectra over the respective chromatographic peak. In addition, to simulate Ex and Em spectra of nonfractionated spent dialysate, total fluorescence Ex and Em spectra were calculated on the basis of a typical 3D fluorescence chromatogram as the sum of all time-point spectra over the whole chromatogram up to the chromatographic retention time when the column washing peaks started to elute, the latter system peaks were excluded.

The relative contribution (RC) of the i-th chromatographic peak (presumably solute) in relation to the total fluorescence of all peaks on a chromatogram, excluding the system peaks, was calculated as a ratio of the area of the i-th peak (A_peak i_) to the total area of all peaks that appeared on the chromatogram (A_total_):

$${\text{RC}}_{\text{i}}\left(\text{\%}\right)=\;\frac{{\text{A}}_{\text{peak i}}}{{\text{A}}_{\text{total}}}\times 100\text{\%,}$$ (1)

The reduction ratio (RR) of a specific i-th peak (uremic solute) for a dialysis session was defined as:

$${\text{RR}}_{\text{i}}\left(\text{\%}\right)=\;\frac{{\text{A}}_{\text{start i}}-{\text{A}}_{\text{end i}}}{{\text{A}}_{\text{start i}}}\times 100\text{\%},$$ (2)

where A_start i_ and A_end i_ are the start and the end HPLC peak areas of the samples from the dialysis session, respectively.

Student’s t-test was used to compare the means of RC_i_ and RR_i_, and p ≤ 0.05 was considered significant. Correlation analysis (Pearsons R) was used for estimation of the degree of linear association between the variable groups of RC_i_ and RR_i_, R > 0.8 was considered as strong correlation. Coefficient of determination (R^2^) was calculated to assess goodness of fit between parameters.

## Results

Fig 1 shows an example of a fluorescence signal of spent dialysate monitored online during a hemodialysis session where fluorescence (Ex: 280 nm, Em: 360 nm) is plotted against the time. The fluorescence drops and peaks during the dialysis correspond to the self-tests in the dialysis machine. The on-line curve reflects elimination of the main fluorophores from blood into spent dialysate.

**Fig 2:**
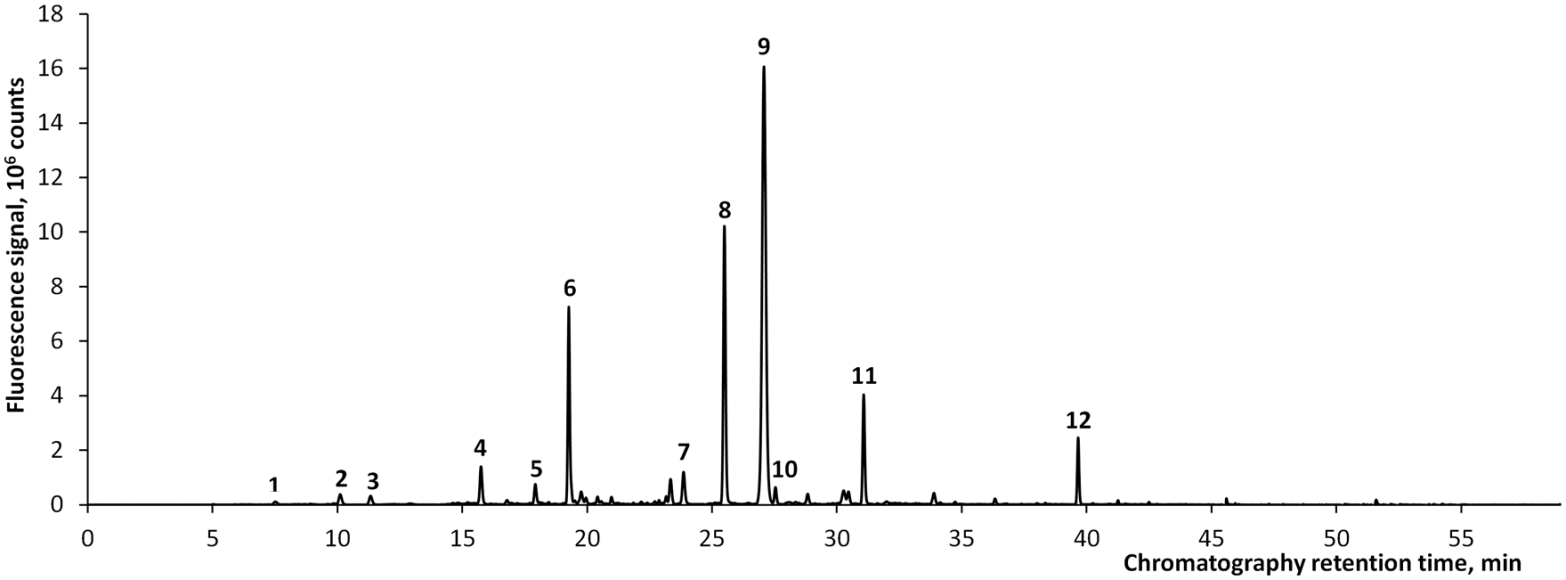
Example of a chromatogram of a spent dialysate sample.

Fig 2 gives an example of a HPLC chromatogram of a spent dialysate sample taken 10 min after the start of hemodialysis, where the fluorescence was recorded at Ex: 280 nm and Em: 360 nm. In total, 12 clearly resolved chromatographic peaks of fluorophoric compounds were detected in most (82%) of the spent dialysate samples during the HPLC analysis (Fig 2) collected at different time moments in the dialysis. Of these, 5 peaks had a major importance in all samples (peaks no 6, 8, 9, 11 and 12), and 6 of these 12 peaks were identified as Tryptophan (Trp) and their metabolites of indole derivates: Indoxyl glucuronide (IGluc), Indoxyl sulfate (IS), 5-hydroxy-indole-3-acetic acid, Indoleacetyl glutamine (IaG), and Indoleacetic acid (IAA) (Table 3).

**Table 3:** Characteristics of the main chromatographic peaks.

Retention time, min		Peak name	Ion mass, negative mode	Ion mass, positive mode
**6**	19.54	Unknown 1	303.08; 365.14	367.15; 389.12
**7**	23.57	Indoxyl glucuronide (IGluc)	308.08	-
**8**	25.62	Tryptophan (Trp)	203.08	205.10; 227.08
**9**	27.17	Indoxyl sulfate (IS)	212.00	-
**10**	27.71	5-hydroxy-indole-3-acetic acid	146.06; 190.05	-
**11**	31.23	Indoleacetyl glutamine (IaG)	302.12	304.13; 326.11
**12**	39.91	Indoleacetic acid (IAA)	130.07; 174.06	176.07

The protein-bound uremic toxin IS appeared to be the major contributor to the fluorescence signal (Table 4) in most of the spent dialysate samples. About a third of the fluorescence signal from the spent dialysate originates from IS. The second contributor to the fluorescence signal in the spent dialysate was Trp, which contributes about a fifth to the signal. The mean contribution of each other fluorophoric compound remained below 10%. Compounds Trp, Unknown 1, and IaG had significantly different (p < 0.05, N = 99) contribution values in the start compared to the contributions in the end of the dialysis. The group of All Other, which consists of the sum of tens of other small peaks, was found to contribute a third to the total fluorescence signal of the spent dialysate in average.

**Table 4:** Contribution (Mean ± SD, Min, Max, N = 99) in percentage for main fluorescent peaks.

	Mean contribution ± SD	Min	Max
Unknown 1	6 ± 3	2	16
Tryptophan	18 ± 8	3	42
Indoxyl sulfate	35 ± 11	5	57
Indoleacetyl glutamine	2 ± 2	0	8
Indoleacetic acid	4 ± 4	1	31
All Other	31 ± 11	10	90

**Fig 3:**
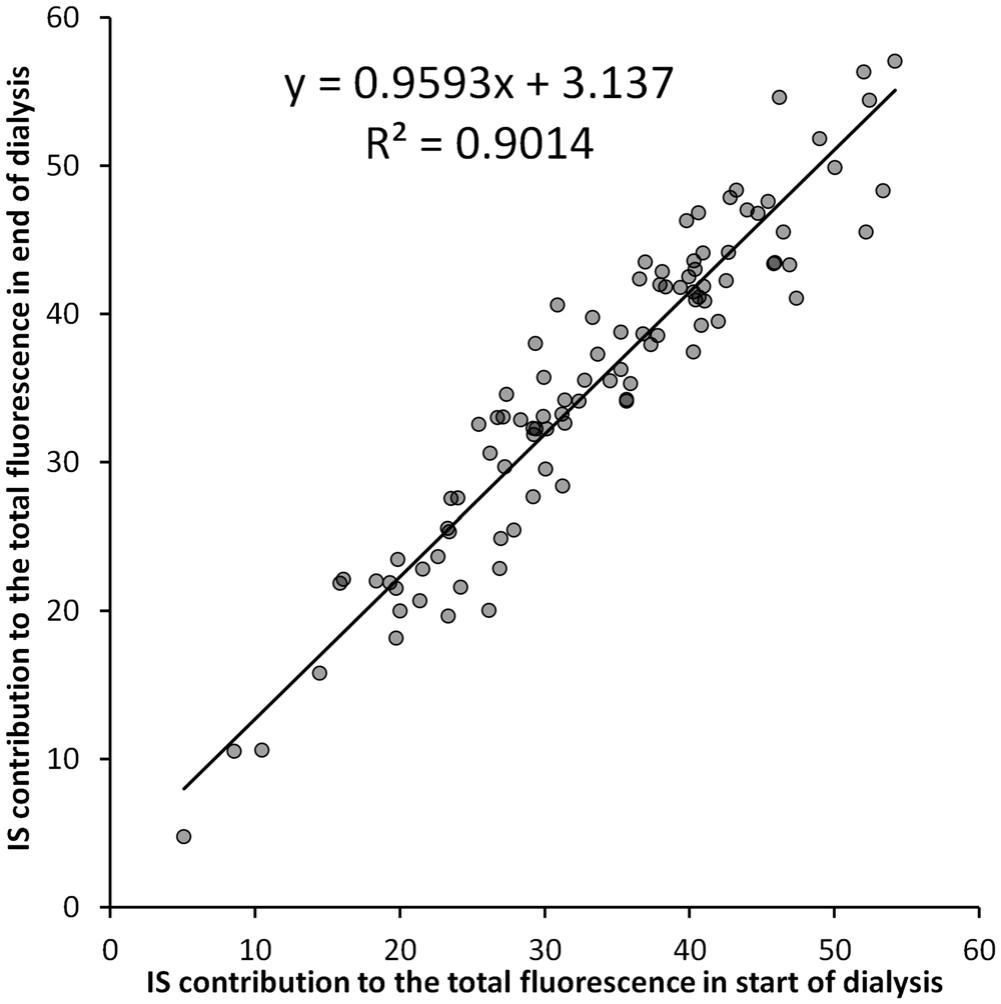
Correlation between the contributions of IS in the Start and End of dialysis.

A comparison of the samples, taken from the start and the end of the dialysis session, was made (Fig 3), to clarify whether the contribution of IS to the total fluorescence is stable in the spent dialysate during the course of the dialysis. The analysis revealed no statistical difference (P = 0.30, N = 99) in the contributions of IS in the samples taken from the start and the end of the dialysis session. Also there was a strong correlation (R^2^ = 0.90, p < 0.001) between these values (Fig 3).

Mean contributions were calculated for 4 identified compounds and 1 unknown solute with the largest contribution. The contribution of 2 identified peaks, IGluc and 5-hydroxy indole-3-acetic acid, was below 1% in most of the cases and therefore these compounds are not described separately. All the other peaks with minor importance or with rare appearance were grouped together as “All Other”.

**Fig 4:**
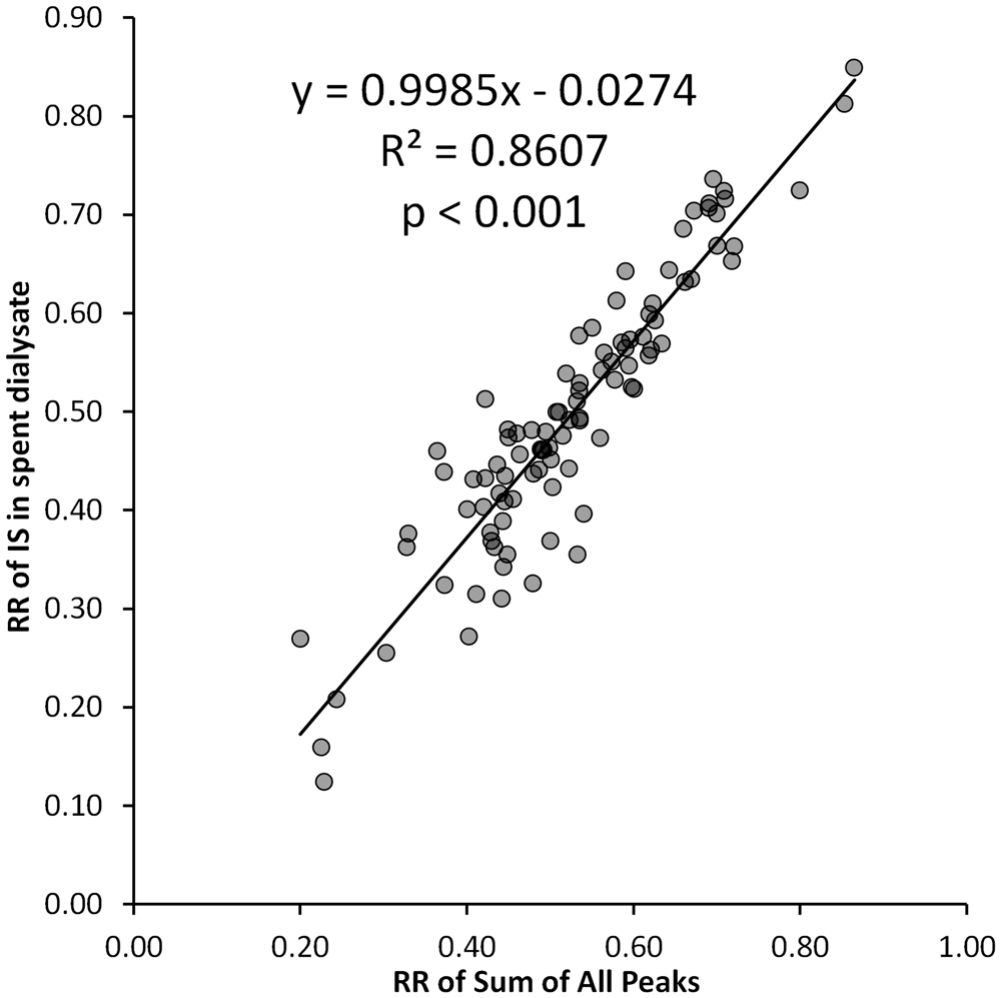
Correlation between the RR of IS and Sum of All Peaks in spent dialysate.

The mean reduction ratio (RR) for the IS, as the main contributor of the fluorescence signal, was 49%, which is very close to the mean decrease of the Sum of All Peaks 51% (P = 0.24) (Table 5) There was a strong correlation (R^2^ = 0.86) between the RR of IS and RR of All Peaks (Fig 4). Trp had very sparse RR with a mean value of 9% and a standard deviation of 24%. It should be noted that during the dialysis, the peak of Trp increased for nearly half of the sessions, resulting in the negative RR values for Trp in these sessions. Peaks of Unknown 1 and IaG had the highest RR values, having close mean values (77 and 79%, respectively), and similar standard deviation, minimal and maximal values. RR of IAA followed with the mean value of 61%, which was higher than the RR of IS (49%, P < 0.05). Large difference between the minimum and maximum and mean RR values for the peaks and the groups indicates large variances in the removal of different fluorophores among the patients and the dialysis sessions.

**Table 5:** Reduction ratio (Mean ± SD, Min, Max, N = 99) in percentage for fluorescent peaks.

Peak name	Mean RR± STDev	Min	Max
Unknown 1	77 ± 10	31	94
Tryptophan	9 ± 24	-71	71
Indoxyl sulfate	49 ± 14	12	85
Indoleacetyl glutamine	79 ± 12	31	95
Indoleacetic acid	61 ± 13	29	89
All Other	31 ± 11	10	82
Sum of All Peaks	51 ± 13	20	87

**Fig 5:**
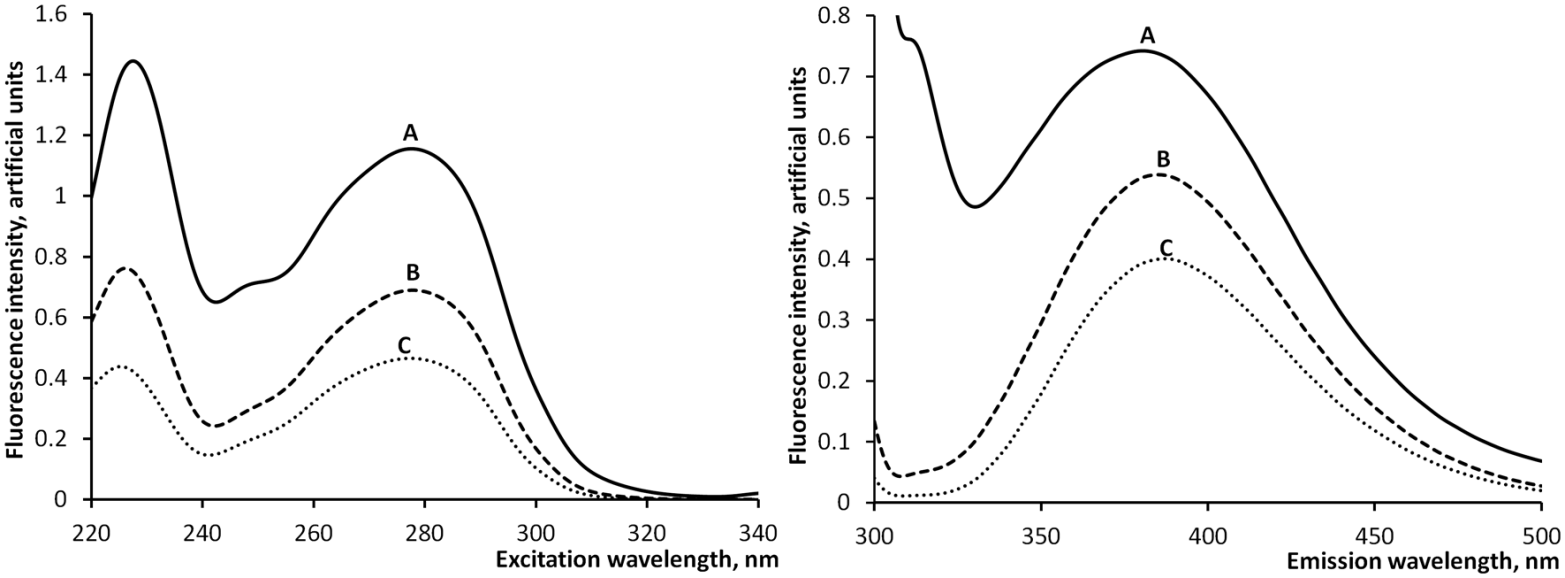
Comparison of Ex and Em scan spectra between the selected peaks and the whole chromatogram. (A)–total fluorescence, (B)–sum of four identified indoles, (C)–IS.

**Fig 6:**
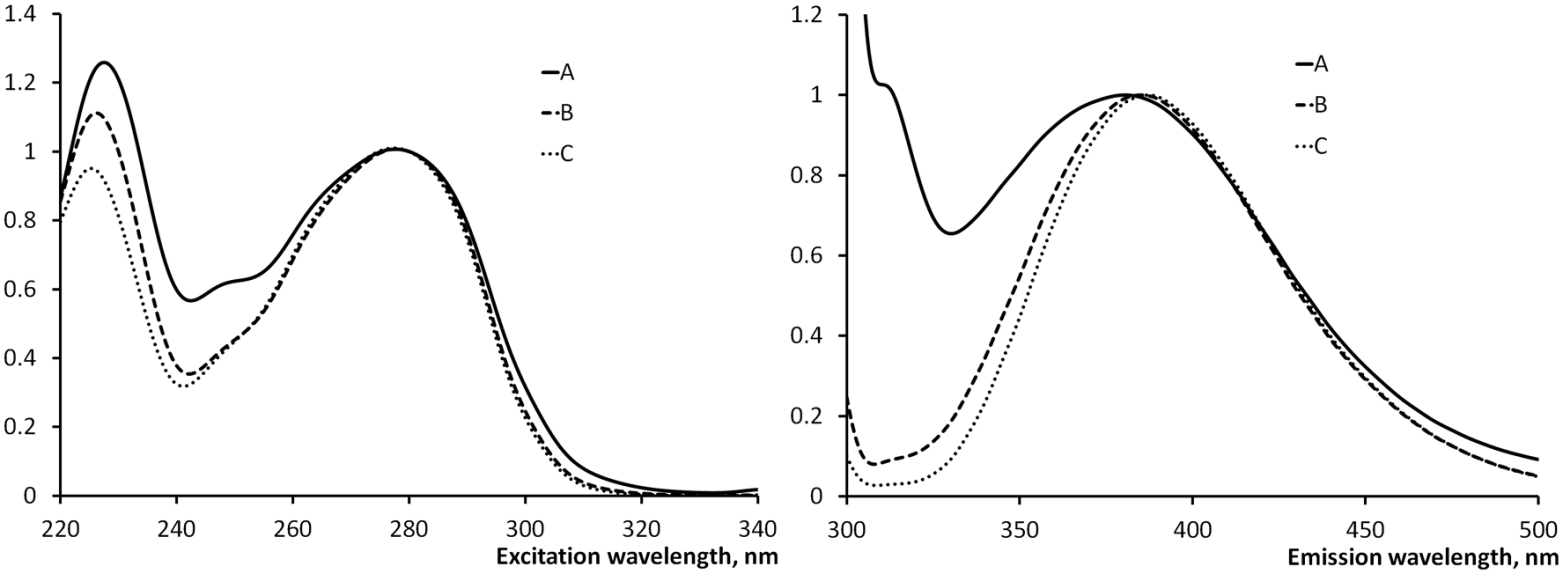
Comparison of normalized Ex and Em spectra between the selected peaks and the whole chromatogram. (A)–total fluorescence, (B)–sum of four identified indoles, (C)–IS.

Figs 5 and 6 summarize the results of the 3D fluorescence chromatograms (N = 10) with an example of a characteristic spent dialysate sample. In Fig 6 Ex spectra were normalized to their values at 280 nm and Em spectra were normalized to their values at 383 nm. These figures show the similarities between the Ex and Em spectra of the total fluorescence of the spent dialysate and the summary spectra of identified indoles (Trp, IaG, IAA, IS). Fig 5 illustrates the contribution level of the IS and four identified indole derivatives to the total fluorescence signal. The sum contributes to more than half of the total fluorescence. Fig 6 presents very similar Ex and Em spectra shapes for the total fluorescence signal, fluorescence of four identified indoles and IS.

## Discussion

Fig 1 presents continuous and on-line fluorescence measurements during a dialysis treatment. This demonstrates first time, by our knowledge, a potential for real-time dialysis monitoring using fluorescence of spent dialysis reflecting removal of the main fluorophores from blood into spent dialysate.

Focus in this study was on the composition of the fluorescence signal measured in the spent dialysate and the estimation of the contribution and removal dynamics of the detected fluorophores. The results aim to consolidate our approach proposed for monitoring the removal of IS [16]. The study indicates that:

1. a predominant part of the fluorescence signal originates from tryptophan and other indole derivatives (IGluc, IS, 5-hydroxy-indole-3-acetic Acid, IaG, and IAA);
2. the highest contribution (35 ± 11%) was found to arise from IS with strong correlation between contribution values at the start and end of dialysis (R^2^ = 0.90);
3. the Reduction Ratio (RR) of IS is very close to the RR from the total fluorescence of spent dialysate (sum of all peaks);
4. very similar fluorescence Ex and Em spectra of the spent dialysate samples from the total fluorescence signal, peaks of 4 identified indoles and peak of IS confirm that the foremost part of the fluorescence signal at used wavelengths originates from the indole derivatives;
5. the former leads to a conclusion that the fluorescence signal at a certain wavelength region (e.g. Ex: 280 nm and Em: 360 nm) has high potential to be utilized for monitoring the removal of PBS IS.

The HPLC results demonstrate that the major fluorophores in the spent dialysate at Ex: 280 nm and Em: 360 nm are indole derivates, PB uremic toxin IS being the main contributor in these measuring conditions (Fig 2 and Table 4). This is in agreement with the earlier studies, where IS has been found as a main contributor to the fluorescence in uremic fluids [18, 19]. Other identified fluorophores in the spent dialysate are IGluc, Trp, 5-hydroxy-indole-3-acetic acid, IaG, and IAA. The Unknown 1 fluorophore remained unidentified, however the fluorescence spectra, relative retention times and ESI-MS positive and negative mode mass-spectra (Table 3) indicate to the possible identification as a tryptophan glucoconjucate [20, 21]. Also, the fluorescence Em and Ex spectra scans indicate that the majority of the fluorescence signal originates from solutes with indole-alike fluorescence spectra with Ex maximum at 280 nm and Em maximum between 350 and 400 nm (Figs 5 and 6). The same fluorescence wavelengths for indoles have been published by others [22]. Therefore it can be assumed that the fluorescence of spent dialysate at Ex: 280 nm and Em: 360 nm is related to a patient’s metabolic status of Trp, an essential amino acid in tight metabolic relation between the human being and the body and is concurrent with guts microflora.

The contribution of IS in the spent dialysate for the samples taken from the start and the end of the dialysis has no statistical difference (P = 0.30) and there is a strong correlation of contribution values between the start and end (R^2^ = 0.90, P < 0.001). This indicates that the contribution of IS to the fluorescence signal does not have significant difference in the start and at the end of dialysis and other fluorophores mutually compensate some differences in the removal rates (Table 4). This is also supported by the very similar RR of IS and the total fluorescence signal in the spent dialysate (Table 5) and strong correlation (R^2^ = 0.86) between these RR values (Fig 4).

The online optical monitoring technology for estimating the dialysis adequacy by the absorbance of UV-light in the spent dialysate was developed more than a decade ago [23]. It has been successfully implemented in everyday clinical practice by integrating the optical sensor into the dialysis apparatus [24]. Lately, the phenomenon of UV-absorbance in the spent dialysate was investigated with HPLC, attributing the largest part of the UV-absorbance signal to the small water-soluble uremic toxin of uric acid rather than to the urea itself [25, 26]. The latter also indicates that the contribution of PBS to the UV-absorbance in the spent dialysate is negligible and the technology based on UV-absorbance monitoring is unsuitable for estimating the removal of PBS. IS has been proposed to serve as a useful indicator for the assessment of the dialysis efficiency (clearance) for PBS beside the urea based dose estimation [27, 28]. Several HPLC methods have been developed and proposed to estimate the removal of IS as a marker for protein-bound uremic toxins [12, 29]. In contrast, the online monitoring of the fluorescence of the spent dialysate assumes no sophisticated HPLC apparatus with specialized laboratory and personnel to estimate the removal of IS. Moreover, fluorescence measurements enable the estimation of the amount of total removed IS [16]. Similarity of the fluorescence spectra of whole dialysate with those of main fluorophores strongly suggests (Fig 6), that monitoring of the fluorescence of the spent dialysate in selected wavelength area characteristic to indole derivatives can be used as a supplementary technology for the assessment of the dialysis efficiency (clearance) of a PBS IS. Although strong positive correlation (R^2^ = 0.84) between RR of IS and other PBS of p-cresyl sulfate has been demonstrated [30] and similar fluorescence detection has been used for analysis of p-cresyl sulfate [31], the fluorescence at Ex: 280 nm and Em: 360 nm is selective only to the indole derivatives and does not cover p-cresol derivatives with different fluorescence wavelength ranges.

A limitation of this study is that it does not yet cover the behavior of main fluorophores in the spent dialysate between the start and the end of dialysis. As it was confirmed by our preliminary results (S1 Fig), the level of fluorophoric dietary amino acid tryptophan had significant fluctuations in concentration in the spent dialysate when the patients had a meal during the dialysis. This effect can be related to the very low mean RR value of Trp (Table 5). Although statistically the contribution of IS remained unchanged during the dialysis, the fluctuating level of Trp may have some influence on the balance of the contribution from the fluorophores to the total fluorescence and on the monitoring accuracy of the removal of IS by fluorescence. Therefore, the behavior of Trp during the whole dialysis should be investigated in more detail to understand the possible errors in estimating the removal of PBS optically. Moreover, fluorescence intensity and spectrum shape of the real spent dialysate as compared to those in the chromatographic eluent are likely to have certain peculiarities, as the pH, different ion composition and the presence of the organic solvents vary in different solvents [22, 32], not forgetting possible quenching and matrix effects [22]. This study was not designed to cover the entire rather complex picture. However, it seems to be prospective to develop the optical monitoring method into a comprehensive technology of on-line assessment of dialysis adequacy, addressing both small water-soluble uremic toxins and some PBS like IS.

## Supporting Information

S1 Fig Example of fluctuation of the tryptophan concentration in spent dialysate during dialysis. (TIF)
