# Supplementary figures and images for: Is Fluorescence Valid to Monitor Removal of Protein Bound Uremic Solutes in Dialysis?
Source: PLoS One. 2016 May 26;11(5):e0156541. doi: 10.1371/journal.pone.0156541 (PMC4882071; doi:10.1371/journal.pone.0156541)

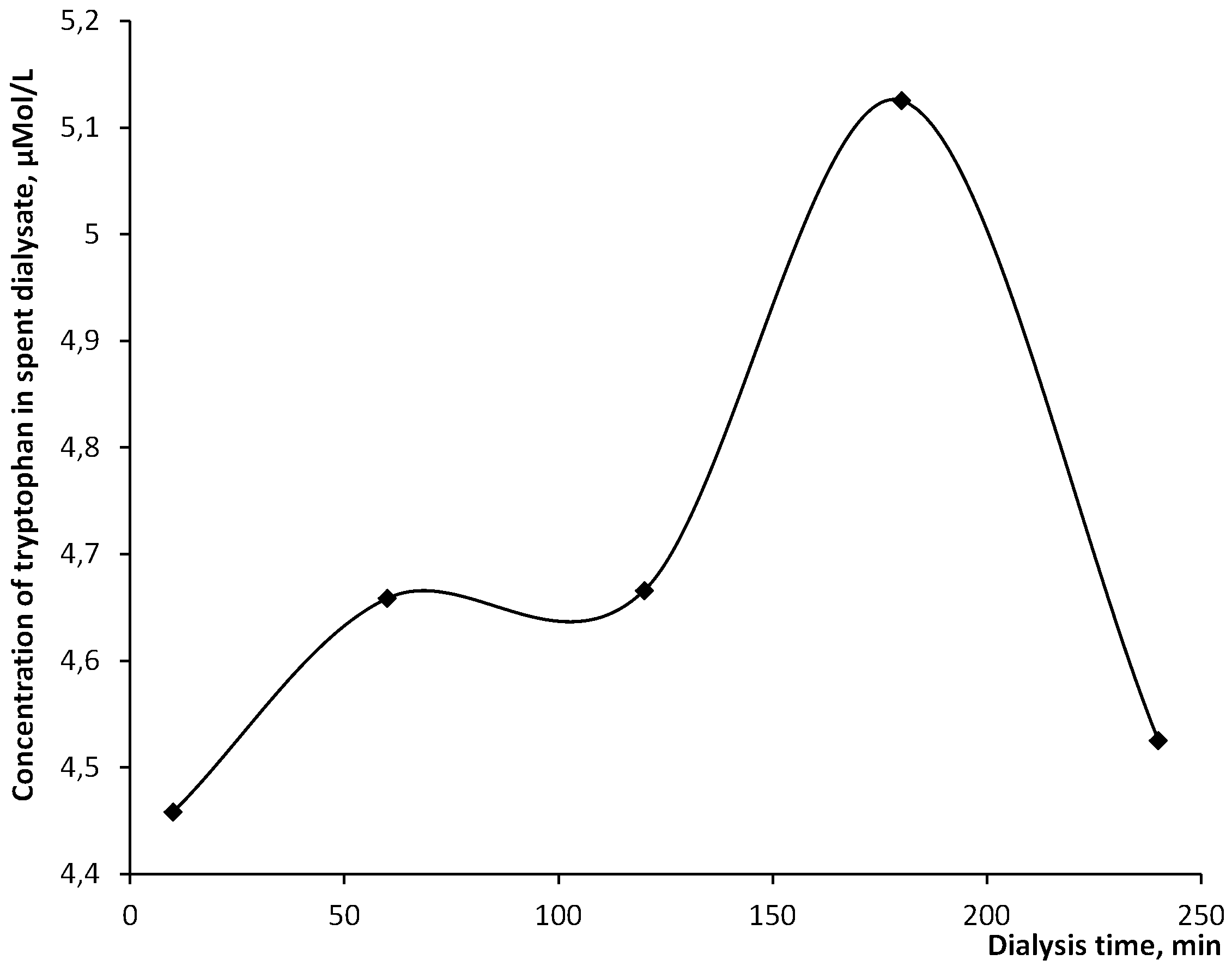

Supplement: S1 Fig — (TIF) [file pone.0156541.s001.tif]
